# Developing and validating teacher formative assessment literacy questionnaire in the high-stakes examination culture: a case of China

**DOI:** 10.3389/fpsyg.2026.1771941

**Published:** 2026-02-11

**Authors:** Rong Fu, Kim Koh

**Affiliations:** Werklund School of Education, University of Calgary, Calgary, AB, Canada

**Keywords:** formative assessment literacy, high-stakes examination context, K–12 teacher development, questionnaire development, teacher assessment literacy

## Abstract

Formative assessment faces significant challenges in its development and implementation among K–12 teachers in Confucian contexts where high-stakes examinations are prominent. To address the need for a validated, culturally grounded instrument to investigate teachers’ formative assessment literacy (FAL), we developed and validated the *Formative Assessment Literacy Questionnaire* (*FALQ*). Drawing on *Conceptual-Practical-Socio-emotional* framework, the research followed a two-phase process: pilot (*N* = 96) and main study (*N* = 309) in China. Exploratory Factor Analysis refined the *Conceptual* dimension into two sub-dimensions, while Rasch analysis identified and removed misfitting items in the *Practical* dimension. Confirmatory Factor Analysis further refined the *Socio-emotional* dimension into two distinct sub-dimensions. The final *FALQ* comprised 25 items across five validated dimensions, demonstrating strong reliability and construct validity. This study contributes a psychometrically robust and culturally adapted instrument for examining FAL in Chinese K–12 settings and offers insights for other Confucian regions.

## 1 Introduction

Formative assessment (FA) has become a crucial and challenging educational facilitator for students’ learning (De Lisle, 2014). In essence, FA has three principles: active teacher-student interaction, an equal partnership between teachers and students, and a focus on improving student learning rather than simply assessing performance (Black and Wiliam, 1998; Yorke, 2003). Simultaneously, to support empirical research in this area, a variety of quantitative survey instruments have been created, revised, and utilized. Such instruments aim to assist scholars, policymakers, and educators in exploring key FA domains, including teacher practices and literacy (DeLuca et al., 2016).

In China, as in other East and Southeast Asian societies, influenced by Confucian Heritage Culture (CHC), FA faces distinct challenges, particularly the dominance of high-stakes examinations and the cultural emphasis on test results (Leong et al., 2018; Yan and Brown, 2021). However, compared to the extensive research and instrument development in Western contexts, such efforts remain minimal in Eastern contexts. This is a significant omission, as Bennett (2011), echoing McMillan et al. (2013), pointed out that teachers’ conceptions about FA are highly diverse and context-dependent, leading to distinct ideas and shaping their practices accordingly.

Thus, a critical research gap exists due to the absence of instruments specifically designed to investigate K–12 teachers’ FA domains within the unique contexts of mainland China, which could also provide insights for other CHC societies with similar culture. Without such tools, researchers cannot adequately examine the distinctive ways FA may be conceptualized, developed and practiced in these contexts. A validated psychometric survey tool is necessary for Chinese scholars to facilitate future research and inform the development of professional development opportunities for teachers by collecting essential evidence. Additionally, studying Chinese teachers’ FA can enrich global and cross-cultural perspectives on FA in Asian CHC contexts. As such, this research drew upon Pastore and Andrade’s (2019) three-dimensional framework to design, develop and validate a teacher questionnaire on FA literacy.

## 2 Literature review

### 2.1 Characteristics of FA

Assessment literacy (AL) has become one of the essential components of teachers to overcome educational assessment challenges in the twenty-first century globally. In general, an assessment-literate teacher (Popham, 2009) should be able to understand and differentiate both “declarative knowledge (‘know-what’) and procedural knowledge (‘know-how’)” (Koh, 2019, p. 21) of educational assessment, which eventually could “influence educational decisions” (Popham, 2011, p. 267). Despite the numerous paradigms, methodologies, and frameworks that constitute the connotation of AL, its practical applications for teachers are primarily separated into two genres: summative assessment (SA) and formative assessment (FA) (Lau, 2016). Koh et al. (2018) explained that the term “literacy” is “used to indicate an individual’s knowledge or competency in a specified subject area” (p. 265). Accordingly, summative/formative assessment literacy (S/FAL) is a sub-concept of AL that refers to a teacher’s understanding and practices of these assessment methods.

SA is a traditional evaluation method that uses rigorous standards, rubrics, or criteria to determine a final score or grade, typically at the end of a learning period, and it is often high-stakes, focusing on a limited number of cognitive outcomes (Lopez and Pasquini, 2017). Although SA is valuable for accountability purposes such as certification, academic ranking, and determining progression, relying on it excessively can lead to “teaching to the test” (Popham, 2001, p. 16) for teachers. As such, scholars such as Black and Wiliam (1998) called for harnessing FA, instead of SA, to hypostatize and promote teachers’ AL in the classroom, as FA has a powerful improvement orientation for student learning (Popham, 2009). As “assessment for learning” is a more comprehensive concept including “formative assessment” (e.g., Bennett, 2011; Klenowski, 2009), we treated them as equivalent terms in the literature and employed the more precise phrase “formative assessment (FA)” herein.

Within the characteristics of FA practice, it is about the *process* of assessment, with an emphasis on the trajectory of assessments rather than the culmination in a product, and it engages students as collaborators and co-navigators within the assessment landscape to improve learning ultimately (Bennett and Gitomer, 2009). In short, FA is a dynamic and ongoing process (Popham, 2009). Second, FA is predicated on *evidence*. The inception of FA, to a certain degree, stemmed from the recognition that teachers were unable to furnish adequate evidence through a singular examination (Stiggins and Chappuis, 2006). Black and Wiliam (2009) advocated that teachers ought to orchestrate a diverse array of assessment relating tasks in practice, such as well-designed classroom mutual dialogues, with the aim of eliciting evidence of student acquisition and achievement. The Assessment Reform Group (2002) pointed out the value of using evidence in education. Teachers and students can seek and interpret such evidence to determine learners’ current position in their educational journey. This information helps them identify the objectives they should pursue and plan the most effective strategies to achieve these goals.

### 2.2 FA dilemma within China

CHC regions commonly refer to China, Japan, Korea, Vietnam, Malaysia, and Singapore, where societies historically influenced by Confucian values, ethics, and social norms (Paramore, 2015). According to Leong et al. (2018) review on FA research, K–12 teachers in the CHC societies are facing complex and significant challenges in implementing FA and developing FAL. Compared to their counterparts in Western classrooms, these struggles stemmed from several fundamental factors, most notably limited FAL (Ratnam-Lim and Tan, 2015). Additionally, in these contexts, high-stakes SA policies, particularly final examinations, remain predominant, e.g., China (Yan and Brown, 2021), Japan (Kuramoto and Koizumi, 2018), Singapore and Malaysia (Koh et al., 2018). Although the current study focused on the context of mainland China, the insights from other CHC regions can shed light on the core dilemmas of implementing FA and developing FAL in mainland China.

Among these factors, policy frameworks play a particularly decisive role in shaping teachers’ opportunities to develop FAL. In terms of legislation, high-stakes summative examinations continue to dominate China’s assessment landscape (Yan and Brown, 2021), though the government has promulgated reforms to reduce this reliance. First, government legislative documents tend to use the Chinese term “评价” (judgment), rather than “评估” (assessment/evaluation) (e.g., Ministry of Education, 2020). This terminology inherently violates the principle of teacher-student parity, a concept fundamental to understanding FA (Heritage and Wylie, 2018). Tan (2018) concluded that, at the policy level, Chinese educational reforms on assessment have had limited success. Ratnam-Lim and Tan (2015) pointed out this is partly because teachers, as policy actors, may respond in diverse ways depending on individual conceptions, skills, attitudes, understanding of the policy’s intent, and willingness to change. Additionally, Liu and Onwuegbuzie (2012) identified three major pressures on Chinese teachers, all tied to SA: high expectations to raise test scores, over-reliance from students and parents for score improvement, and the critical use of examination results in teacher evaluations in school. Consequently, teachers, students, and parents tend to overlook alternative assessment methods but put exclusive emphasis on SA results. Moreover, the dominance of high-stakes examination policies may restrict teachers’ opportunities to develop FAL, as their professional focus remains tied to test development and score improvement.

Beyond policy constraints, traditional socio-cultural influences from Confucianism represented another major barrier in the FA dilemma in China. Ratnam-Lim and Tan (2015) contended that the highly authoritative nature of the teacher-student relationship, rooted in the cultural mindset of respecting teachers, fundamentally conflicts with the equality principle of FA. Originally, it is the filial piety (obedience) from juniors (children/students) to seniors (parents/teachers), a key principle of traditional Confucian values, serves as a socio-cultural barrier (Guo and Cao, 2024). Within this mindset, teachers are often framed as knowledge transmitters through a teacher-centred approach, exemplified by the Virtuoso Model or as gardeners nurturing student growth with a sense of sanctity (Guo, 2005). Additionally, after the Reform and Opening-up in 1978, Socialism with Chinese Characteristics integrated the Confucian concept of *He* (和, harmony). In classroom practice, this often manifested as conformity, leading to passive student engagement and overlooking individual differences (Tan, 2018). As a result, Chinese teachers often have fewer opportunities to acknowledge and address classroom diversity and provide equitable engagement in FA (DeLuca and Lam, 2014) and undermine the positive attitudes required for FAL.

Lastly, in Chinese classroom practices, FA initially garnered attention primarily within the domain of English language teaching in the early 2000s and in the context of university (currently, it remains the predominant area of studies about FA in China) (e.g., Xu and Zhang, 2020). Consequently, FA practices and teachers’ FAL in other K–12 disciplines have remained under-researched.

### 2.3 FA survey tools within China

Over the past three decades, numerous quantitative scales, questionnaires, inventories, and other instruments have been developed and validated for researching topics related to AL in Western classrooms (e.g., DeLuca et al., 2016; Plake et al., 1993). These instruments included limited items of FAL as one of the dimensions of AL. Further, DeLuca et al. (2016) argued that most AL instruments are outdated as they were based on early conceptions of AL that did not reflect significant changes in assessment principles, particularly those concerning FA.

Moreover, from a global perspective, few instruments were specifically designed to measure the different domains of FA from the perspectives of K–12 teachers. Among them, Yan and Pastore (2022a,b) developed and validated two FA scales: the *Teacher Formative Assessment Literacy Scale* (*TFALS*) and the *Teacher Formative Assessment Practice Scale* (*TFAPS*). Their findings from K–12 teachers in Hong Kong significantly shed light on FA practices in Asia. A limitation is that these two scales lack items designed specifically for the CHC context. This is because Hong Kong would represent a hybrid context shaped by both Confucian and British educational traditions. Although it is often viewed as an example of CHC and a high-stakes examination region, Hong Kong teachers’ FAL may differ from those in other traditional CHC contexts. This divergence is largely due to the widespread implementation of school-based assessment in Hong Kong, which was introduced to mitigate the dominance of high-stakes examinations (Xiao et al., 2023). Additionally, the items in these two scales had a Westernized wording, making it difficult to adapt them in a way that local teachers in mainland China could easily understand, such as the Western concept of “ethics” and “studnet right,” which may not have a direct equivalent or may be understood differently. Further, teachers’ communication with parents about student learning, another important scenario in Chinese schools regarded as an integral duty of teachers, has not been yet included in previous survey scales either.

Furthermore, in mainland China, there has been limited effort to develop questionnaires for FA. Wang (2017) designed the *English Interpretation Competency Scale* which included elements of FAL but was not designed to measure FAL comprehensively. Simultaneously, Guo and Xu (2021) developed a FA questionnaire specifically for university students’ English as a Foreign Language writing. These two instruments were solely aimed at investigating students’ English proficiency within the university context. Additionally, Zou et al. (2021) designed a Likert-style questionnaire using ICT tools/platforms for FA during the COVID-19 pandemic, which was heavily technology-oriented and lacked cultural elements. Although these instruments cannot be directly applied to survey Chinese teachers’ FAL, they provided valuable methodological references for designing the questionnaire in this study.

## 3 Methodology

The primary aim of this study was to design, develop, and validate a questionnaire on Chinese teachers’ FAL, providing researchers, policymakers and teachers with a reliable investigative tool that has broad applicability (Price and Barrell, 1980). Given that K–12 teachers’ FAL in mainland China remained relatively novel and understudied, the study employed a two-phase design: a pilot study with a small sample to verify the theoretical framework and refine items, and a main study with a larger sample to establish final validity (DeVellis, 2012).

### 3.1 Participants

The participants for the study were K–12 teachers teaching across all subjects from 17 public schools in Lingang, Shanghai, China. Teachers from three schools (one primary, one secondary, and one high school) took part in the pilot study, while teachers from the remaining 14 schools (five primary, five secondary, and four high schools) participated in the main study. This study was approved by the research ethics committee of our affiliated university, and all participants provided informed consents before participating in the survey.

### 3.2 Framework of questionnaire

Drawing on the literature, the *Formative Assessment Literacy Questionnaire* (FALQ) was designed, developed, and validated based on Pastore and Andrade’s (2019) three-dimensional framework consisting of *Conceptual*, *Practical*, and *Socio-emotional* dimensions. The *Conceptual* dimension refers to the conceptions a teacher holds about FA, the *Practical* dimension emphasizes integrating the FA process with teaching practices to monitor, evaluate, and manage the teaching-learning process, and the *Socio-emotional* dimension involves managing the social and emotional aspects of FA. The items were developed and categorized according to keywords aligned with each dimension. The response format of the *FALQ* employed a five-point Likert scale, ranging from 1 (strongly disagree) to 5 (strongly agree). The questionnaire consisted of 30 items, with 10 items allocated to each of the three dimensions (see Supplementary Appendix). In addition to the main body of the *FALQ*, two items were designed to collect participants’ demographic information about school type and teaching subject.

### 3.3 Review panel

To ensure content validity, a panel of four experts, including two professors in psychometrics and two Chinese K–12 teachers (one experienced and one novice), reviewed the questionnaire. They evaluated the clarity, relevance, and wording of the items, as well as the participant instructions. Each item was rated on a four-point scale: 0 (needs removal/replacement), 1 (needs major revision), 2 (needs minor revision), or 3 (no revision needed) based on two criteria: (1) clarity and (2) essentiality. Additionally, both in-service teachers confirmed that the Chinese edition of the questionnaire is easily readable for regular in-service teachers.

For clarity, the average scores of items in the *FALQ* were 2.81 out of 3, while for essentiality, the averages were 2.93 out of 3, indicating strong overall quality. The majority of items achieved the highest rating (3 points), with no items deemed unsuitable (0 points). The items scoring 1 (requiring major revision) were prioritized for refinement, either through rewording or scenario adjustments, thereby enhancing the validity of the instrument. This procedure can strengthen the clarity, relevance, and validity of the *FALQ* before the pilot study.

## 4 Results of the pilot study

### 4.1 Descriptives

The online link of the questionnaire on Qualtrics was distributed to approximately 160 teachers across three schools. Of these, 101 teachers completed the questionnaire. After removing missing or invalid responses, such as incomplete submissions or perfunctory responses where the same answer was given for all items, 96 valid responses remained for analysis. The participants (*N* = 96) included 31 primary school teachers, 33 secondary school teachers, and 32 high school teachers. Most school subjects were covered: Chinese Language (*N* = 26), English (*N* = 28), mathematics (*N* = 17), sciences (e.g., physics) (*N* = 12), social sciences (e.g., history) (*N* = 9), and arts and physical education (*N* = 4). The mean *FALQ* score was 118 (SD = 15.17) out of a maximal score of 150 (78.67% scoring rate).

### 4.2 Reliability

According to Nunnally and Bernstein (1994), Cronbach’s alpha coefficient value of 0.70 was deemed to be acceptable for reliability in survey research. In this study, the Cronbach’s alpha for *FALQ* was ideal (= 0.90). Thus, the internal consistency reliability of the questionnaire data provided a solid foundation for the subsequent analyses.

### 4.3 Exploratory factor analysis of FALQ

Although the questionnaire structure and items were based on the Conceptual–Practical–Socio-emotional framework, it was necessary to conduct an Exploratory Factor Analysis (EFA) to distill the data and obtain a more accurate picture of the factors first, as the framework remained brief and insufficiently supported by empirical studies. This analysis is crucial for assessing the construct validity of the questionnaire (Fabrigar and Wegener, 2012). A small sample size of at least 50 and no more than 100 subjects is generally considered adequate for conducting EFA (Sapnas and Zeller, 2002). Additionally, while some statisticians have suggested various rules for the subject-to-item ratio, such as 3:1, Hogarty et al. (2005) pointed out that there is no specific minimum ratio required to achieve valid outcomes.

The data analysis software was Mplus 8.10 (Muthén et al., 2018). The value for Kaiser–Meyer–Olkin of Sampling Adequacy (KMO) was 0.905, and the Bartlett’s Test of Sphericity showed χ2 (96) = 3601.364, *p* = 0.000. A KMO index greater than 0.900 is considered highly suitable for factor analysis, while Bartlett’s Test of Sphericity should be significant (*p* < 0.05) (Williams et al., 2010). Therefore, the data of *FALQ* were suitable for EFA.

The EFA was conducted using maximum likelihood extraction with oblique rotation. The EFA yielded four latent factors (see Table 1). Factor retention was determined primarily through parallel analysis, which supported a four-factor solution, and was further corroborated by eigenvalues > 1.0. Item evaluation was based on factor loadings, communalities, and cross-loadings to ensure a clear and interpretable factor structure. Items were retained if they demonstrated a primary factor loading of at least 0.45 and were conceptually aligned with the corresponding factor. In addition, all items exhibited substantial communalities, ranging from 0.637 to 0.891, exceeding the commonly recommended threshold of 0.40 (Hair et al., 2018). These results indicated that a substantial proportion of variance in each item is explained by the extracted latent constructs.

**Table 1:** *FALQ* items’ factor loadings across four dimensions.

	Factors				
Items	Teachers’ self-efficacy	Knowledge for collaborating with students	Practical	Socio-emotional	Communality (*h*^2^)
Q1	0.822				0.831
Q2	0.815				0.837
Q3	0.650				0.768
Q4	0.718				0.766
Q5		0.619			0.708
Q6		0.727			0.749
Q7		0.725			0.736
Q8		0.748			0.876
Q9		0.756			0.891
Q10		0.644			0.820
Q11			0.532		0.694
Q12			0.535		0.719
Q13			0.648		0.850
Q14			0.657		0.714
Q15			0.740		0.797
Q16			0.797		0.783
Q17			0.625		0.800
Q18			0.479*	0.538*	0.759
Q19			0.571		0.829
Q20			0.577		0.803
Q21				0.758	0.822
Q22				0.712	0.754
Q23				0.734	0.637
Q24				0.743	0.789
Q25				0.807	0.851
Q26				0.755	0.823
Q27				0.710	0.775
Q28				0.782	0.736
Q29				0.791	0.810
Q30				0.794	0.847

*Indicates item with cross-loadings.

Within the *Conceptual* dimension, the items clustered into two sub-dimensions based on their factor loadings and thematic content: (1) *Teachers’ self-efficacy* and (2) *Knowledge for collaboration with students*. Bandura (1997) explained that self-efficacy represents a psychological construct distinct from fundamental knowledge, while Black and Wiliam (2009) emphasized the critical role of teachers’ knowledge in fostering collaborative practices. This evidence supported distinguishing the two sub-dimensions within the broader *Conceptual* domain. Further, most of the items indicated significance in EFA, except Q18 *Facilitating peer feedback* (the only item showing a notable cross-loading), which was slightly controversial as it indicated similar values in the *Practical* dimension (= 0.479) and the *Socio-emotional* dimension (= 0.538). This item was about facilitating peer feedback, which was originally categorized in the *Practical* dimension. Upon scrutiny, Q18 *Facilitating peer feedback* was a practical question but can also be viewed as a question of sociocultural perspective, as peer feedback largely depends on factors such as the teacher’s role in facilitation and students’ attitudes (Ng and Yu, 2021). The *Practical* dimension included items focused on assessment activities with specific actions (Q18 used the key verb “facilitate”), whereas the *Socio-emotional* dimension comprised items reflecting abstract psychological concepts. Finally, to maintain consistency, it was better to place Q18 *Facilitating peer feedback* into the *Practical* dimension. Additionally, the dimension comprising items Q11–Q17, Q19 and Q20 aligned with the *Practical* dimension, and items Q21–Q30 consistently fell within the *Socio-emotional* dimension.

### 4.4 Rasch analysis

**Figure 1:**
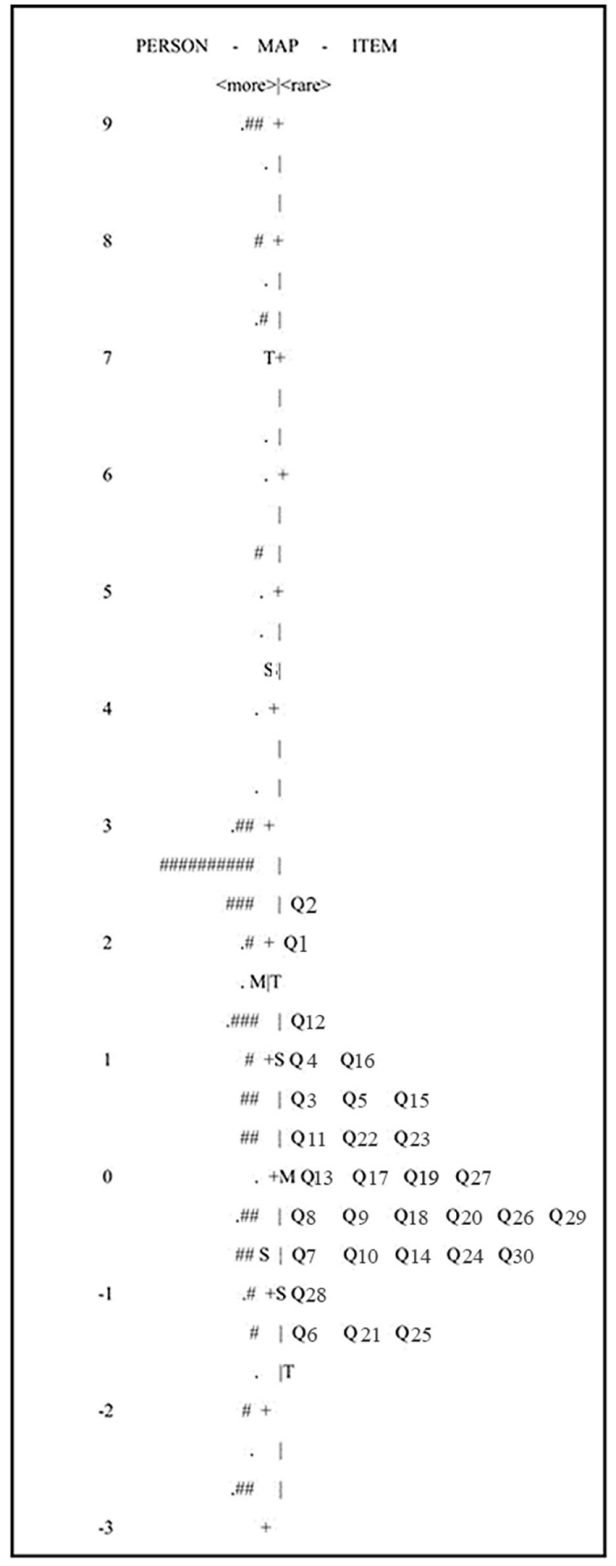
The distribution of item-person map of *FALQ*. Each “#” is 2 and each “.” is 1.

To verify the validity of psychometric research scale, the Rasch Model is often harnessed as one primary method in the related fields. In this study, we used the software Winsteps (Linacre, 2024) for the Rasch analysis of the *FALQ* at the overall scale level. Rasch analysis supported essential unidimensionality of the overall scale as indicated by Principal Component Analysis of Residuals, with the first contrast eigenvalue < 2.0. This suggests that the four dimensions identified through EFA are underpinned by a common latent construct. Additionally, local independence was confirmed, as all item residual correlations were < 0.30 (Linacre, 2024). Figure 1 presented the item-person maps, displaying person measures and item difficulties on a logit scale, with logit positions indicated on the sides like a ruler. All logit positions ranged from −2.5 to +3, within the acceptable range of −3 to +3 (Engelhard and Wang, 2021), indicating an appropriate spread of item difficulty and person ability along the latent continuum. This distribution suggests that the scale functions well across the targeted range of the construct. Figure 1 also showed that, while the distribution of persons’ FAL scores was wide, item difficulties were slightly skewed toward low to moderate levels, with limited coverage for higher FAL scores. This likely reflects both the characteristics of the pilot sample and the preliminary stage of the questionnaire, a phenomenon commonly observed in pilot testing (Bond et al., 2020). The rating scale functioning was validated by ordered category thresholds, showing a monotonic increase in difficulty across response options. Overall, the alignment between person ability and item difficulty (ranging primarily from −2.5 to +3 logits) indicates good targeting and minimal standard error for the target population.

The results of *FALQ* well fitted to the Rasch Model (Table 2): Infit MNSQ = 0.97; Outfit MNSQ = 0.89, which fall within the acceptable range of 0.5 to 1.5 (Bond et al., 2020). Each item’s measure value represented its level of difficulty. The total logits for *FALQ* (= 0.27) were positive and above zero, indicating that the overall difficulty was appropriate and capable of effectively assessing the participants’ actual proficiency (Andrich, 1988). However, some items indicated misfit: Q13 *Feedback for different scenarios* (= 0.48), Q17 *Further suggestions* (= 0.46), Q18 *Facilitating peer feedback* (= 0.44), Q19 *Identifying strengths & weaknesses* (= 0.41) and Q20 *Comparing student own performances* (= 0.31) were slightly lower than the acceptable range. As such, we excluded these items from the main study. In summary, the original questionnaire items generally fitted the Rasch model, although several items showed misfit. Given that it was a pilot study conducted in an uncharted area with a small sample size, the result of Rasch Analysis can still be considered acceptable (Bond et al., 2020).

**Table 2:** *FALQ* item Rasch analysis with outfit and infit statistics.

Item	Item measure	Infit MNSQ	Outfit MNSQ
Q1	2.02	1.25	1.33
Q2	2.23	1.37	1.42
Q3	0.71	1.17	1.26
Q4	1.16	1.37	1.43
Q5	0.51	1.21	1.12
Q6	−1.20	1.25	1.42
Q7	−0.66	1.48	1.49
Q8	−0.28	0.81	0.60
Q9	−0.35	0.86	0.67
Q10	−0.58	0.67	0.51
Q11	0.23	1.00	0.79
Q12	1.22	1.14	1.04
Q13	0.02	0.66	0.48
Q14	−0.66	1.22	1.57
Q15	0.58	1.07	0.91
Q16	0.91	1.14	1.06
Q17	−0.13	0.55	0.46
Q18	−0.43	0.61	0.44
Q19	−0.13	0.51	0.41
Q20	−0.43	0.52	0.34
Q21	−1.27	0.65	0.58
Q22	0.30	0.88	0.67
Q23	0.30	1.51	1.21
Q24	−0.66	0.78	0.61
Q25	−1.2	0.80	0.79
Q26	−0.28	0.81	0.71
Q27	0.16	0.80	0.58
Q28	−1.12	1.24	1.25
Q29	−0.35	0.90	0.70
Q30	−0.66	0.80	0.71
Total	0.27	0.97	0.89

All measures are in logits.

## 5 Results of the main study

### 5.1 Descriptives

In the main study, the revised *FALQ* was distributed via a Qualtrics online link to approximately 600 teachers across 14 public K–12 schools in Lingang. Of these, 411 teachers completed the questionnaires (65% participation rate), indicating a good return rate for online survey. However, after cleaning for erroneous, missing, or incomplete responses, 309 valid responses (*N* = 309) remained for analysis. The inclusion criteria required responses to be complete and submitted within an appropriate time frame of at least 10 min, reflecting a normal pace and helping to avoid perfunctory responses. Among them, 100 participants were from primary schools, 128 from secondary schools, and 81 from high schools. The participants taught a range of K–12 subjects, including Chinese Language (*N* = 76), English (*N* = 59), mathematics (*N* = 55), sciences (e.g., physics) (*N* = 40), social sciences (e.g., history) (*N* = 40), and other subjects (fine arts, physical education, computer science) (*N* = 39), representing the primary subjects taught in Chinese K–12 schools.

The mean overall score for *FALQ* was 102.55 out of 125 (SD = 13.69). By extension, the mean scores for each factor within the *FALQ* were as follows: *Teachers’ self-efficacy*: 15.03 out of 20 (SD = 3.096), *Knowledge for collaboration with students*: 25.25 out of 30 (SD = 3.617), *Practical*: 20.42 out of 25 (SD = 3.042) and *Socio-emotional*: 41.84 out of 50 (SD = 13.686).

### 5.2 Reliability

In the main study, Cronbach’s alpha coefficient for the *FALQ* was ideal (*α* = 0.877), closely aligning with the pilot study result.

### 5.3 Confirmatory factor analysis of FALQ

The value for Kaiser–Meyer–Olkin of Sampling Adequacy (KMO) was 0.856, and the Bartlett’s Test of Sphericity showed χ2 (309) = 1398.195, *p* = 0.000, indicating that *FALQ* was suitable for Confirmatory Factor Analysis (CFA). The initial findings of CFA for the four-factor model indicated that some fit statistics were satisfactory: CFI = 0.910 and TLI = 0.900 (≥ 0.9000); however, other statistics were less than ideal: RMSEA = 0.093 (exceeding the ideal goodness-of-fit threshold of ≤ 0.08) and SRMR = 0.064 (exceeding the ideal threshold of ≤ 0.05) (Hu and Bentler, 1999).

To address the suboptimal model fit, the factor structure was refined following a theory-driven and incremental approach. Modification indices were examined to identify areas of misfit; however, no correlated error terms were added, and no parameters were freed solely to improve fit. Across five theoretically plausible alternative models with restricted factor organizations (Chan and Luo, 2021), CFA results consistently suggested that the 10 *Socio-emotional* items reflected two interrelated but distinct sub-dimensions: *Concerning student emotions* (six items) and *Respect for students* (four items). This decision was informed not only by item factor loadings and content themes, but also by socio-cultural perspectives on teacher-student interactions that highlight the importance of emotional responsiveness and student dignity in China (Chan and Luo, 2021; Gan et al., 2019).

**Figure 2:**
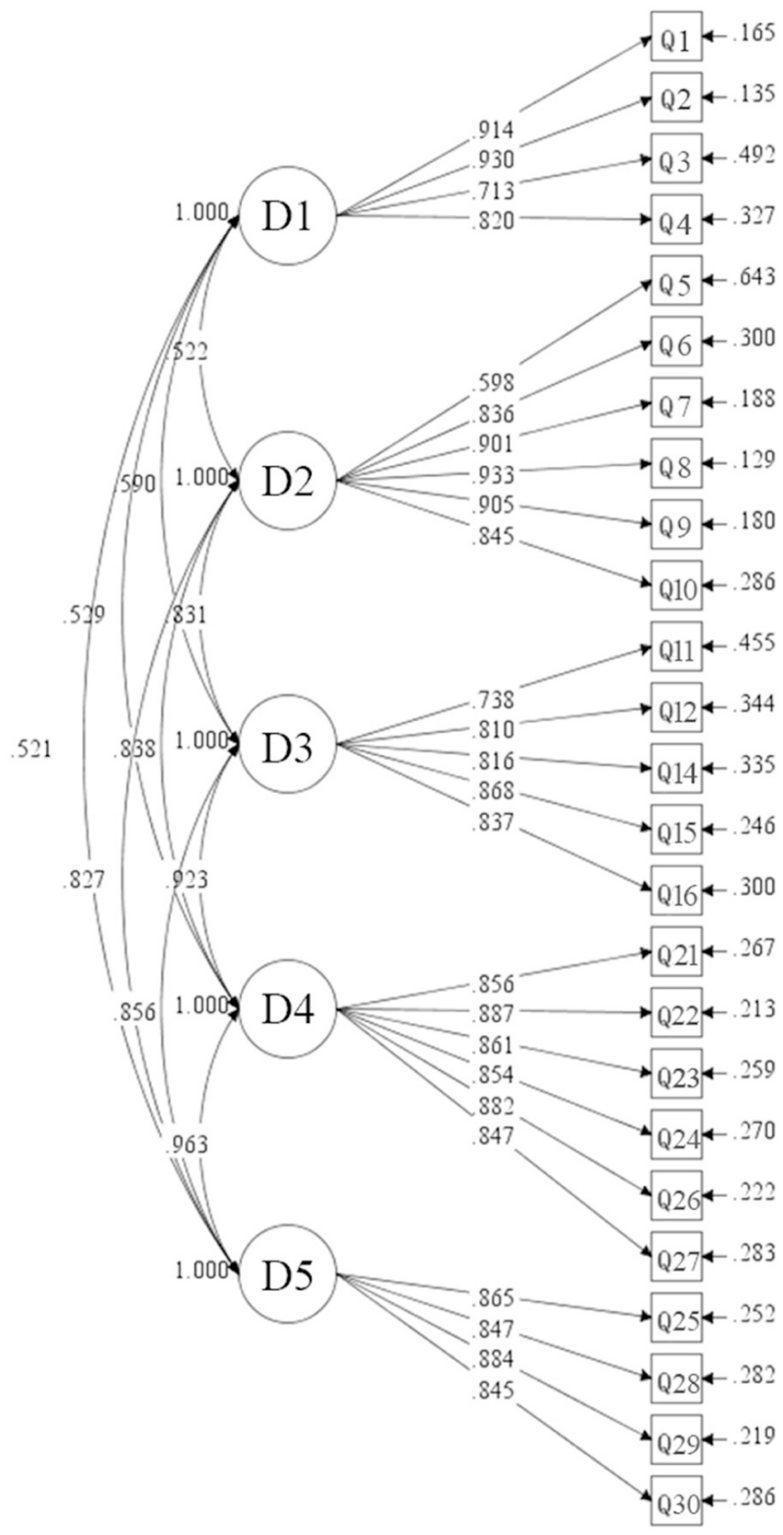
Standardized factor loadings of items in the five-dimension confirmatory factor analysis. D1 = *Teachers’ self-efficacy*; D2 = *Knowledge for collaboration with students*; D3 = *Practical*; D4 = *Concerning student emotions*; D5 = *Respect for students*.

Then we conducted a CFA with this five-factor model: (1) *Teachers’ self-efficacy*, (2) *Knowledge for collaboration with students*, (3) *Practical*, (4) *Concerning student emotions* and (5) *Respect for students*. The outcomes demonstrated improved and acceptable model fit between the hypothesized model and the observed data: RMSEA = 0.078, CFI = 0.926, TLI = 0.916 and SRMR = 0.052. Although the SRMR value (= 0.052) slightly exceeded the common cutoff of 0.05, it remained within an acceptable range when considered alongside other fit indices (Kline, 2010). Additionally, each item indicated a high factor loading in the five-factor model (Figure 2). All factor loadings ranged from 0.598 to 0.933, indicating strong associations with their respective latent constructs (Gorsuch, 2015). Given the improvement in model fit and the enhanced conceptual clarity afforded by distinguishing *Concerning student emotions* and *Respect for students*, the five-factor model was deemed more appropriate than the original four-factor structure. Taken together, the CFA results provided strong evidence for the construct validity of the *FALQ* in the main study.

After revision, the mean scores for the new sub-dimensions were: *Concerning student emotions*: 25.10 out of 30 (SD = 9.4) and *Respect for students*: 16.77 out of 20 (SD = 6.3).

## 6 Discussion

**Figure 3:**
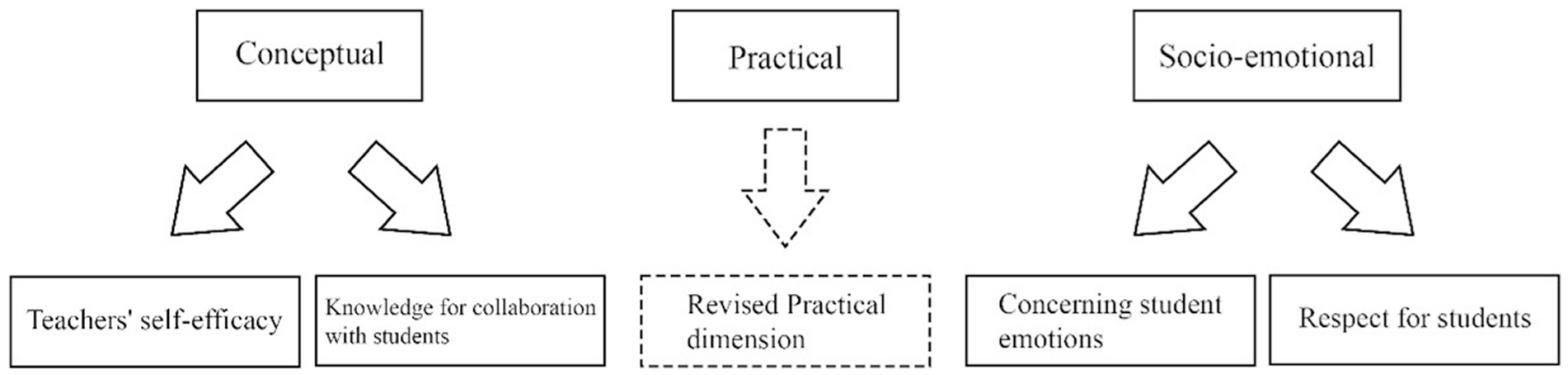
Revised FAL framework expanding from three to five dimensions.

This study designed, validated, and developed a questionnaire instrument for measuring Chinese teachers’ FAL through a pilot-main study procedure. The EFA, CFA and Rasch Analysis demonstrated a clear five-dimensional questionnaire. The final version of the *FALQ* has 25 items accessing five dimensions of FAL, i.e., *Teachers’ self-efficacy* dimension (4 items), *Knowledge for collaboration with students* dimension (6 items), *Practical* dimension (5 items), *Concerning student emotions* dimension (6 items) and *Respect for students* dimension (4 items) (Figure 3). Since Pastore and Andrade (2019) deliberately refrained from ranking original FAL dimensions to allow for cultural adaptability, the revised *FALQ* framework treated the five dimensions as parallel. As Yan and Pastore (2022a) pointed out, to study teachers’ FA, it is essential to establish a valid and user-friendly instrument at first. The final revised questionnaire contributed by extending and refining the theoretical framework of FA (Xu and Brown, 2016), and serving as a valid and practical survey tool for future research on FAL in China and other CHC contexts.

First, in the pilot study’s EFA, we refined the original *Conceptual* dimension into two more specific dimensions: *Teachers’ self-efficacy* and *Knowledge for collaboration with students,* which are the two fundamental components of teacher’s holistic FAL construction (Xu and Brown, 2016). Teachers’ self-efficacy is their belief or confidence in successfully completing FA-related tasks (Hartell et al., 2014). As an important sub-factor within the *Conceptual* domain of AL (Schneider and Bodensohn, 2017), teacher self-efficacy functions as a critical higher-order component of teachers’ conceptions and can influence FA acquisition and practices (Crusan et al., 2016). Yan and King (2023) elaborated that Chinese teachers’ self-efficacy regarding FA is dynamic from a sociocultural perspective. Thus, it is necessary to focus specifically on Chinese teachers’ self-efficacy. Amid the limited evidence in China, Fu (2024) asserted that self-efficacy directly influences Chinese teachers’ FA practices, particularly in the selection and development of FA methods. Additionally, pre-service teachers in China generally lack confidence in engaging students, a factor of self-efficacy closely associated with FAL (Chen, 2019). Moreover, self-efficacy is a fundamental component that can guide teachers’ professional development in China (Ding and Hong, 2024). Therefore, the *Teachers’ self-efficacy* dimension can particularly provide valuable insights for designing future teacher training programs about building confidence as a crucial first step. At the same time, the *Knowledge for collaboration with students* dimension emphasized teachers’ capacity to work with students in an equal and reciprocal manner, eventually to set up a feeling of belonging to Chinese students (Luo et al., 2022). It extended beyond the knowledge base of assessment methods (Xu and Brown, 2016) to include understanding how to engage students as partners in assessment practices. Wafubwa (2020) suggested that teachers should understand how to motivate student to engage in FA practices. However, this poses a particular challenge for Chinese teachers, who should overcome the traditional mindset of hierarchical teacher-student relationships (Chong and McArthur, 2023), and therefore requires greater attention in teacher professional development within Confucian contexts.

Second, the Rasch Analysis in the pilot study identified five items misfitting in the practical dimension: two directly related to feedback practices and three concerning the monitoring of FA processes through feedback. Morris et al. (2021) indicated that feedback can help students recognize mistakes, guide them in improving specific parts of their answers, and encourage reflection on their previous contributions. That is, these five items are conceptually homogeneous, focusing on scaffolding student learning in the classroom (Tabak and Reiser, 2022), as they all require teachers to actively engage in the cascade of FA activities. Although the fundamental principle of FA lies in the utilization of teacher feedback to move learners forward (Black and Wiliam, 2009), resulting in assessment *for* learning, many current Chinese teachers likely have limited preparation and experience in providing feedback. For instance, Zhan (2024) suggested that Chinese pre-service teachers need to develop feedback literacy, as a lack of it may negatively affect their future in-service practices. However, a distinction should be made between general classroom feedback and feedback intended specifically for FA purposes. Although Liu et al. (2025) argued that Chinese teachers are skilled at identifying issues and providing actionable guidance by feedback, there is little evidence in China that examines how FA principles are applied in feedback practices, such as maintaining an equal and reciprocal teacher-student relationship. For instance, Tian and Zhou (2020) asserted that students are encouraged to critically evaluate teacher feedback and make decisions on whether to incorporate it into their learning. Moreover, Saito and Inoi’s (2017) findings from Japanese secondary and high school English classrooms suggested that individual differences in FA-related feedback, such as planning, preparation, and immediate reactions, can pose barriers for teachers. Additionally, prior scale development research in the Chinese context has conceptualized FA and feedback as related but distinct clusters. For example, the *Assessment for Learning Experience Inventory* (Gan et al., 2019) operationalized feedback as a specific instructional practice supporting, but not equivalent to, other factors of FA, and measured the two at differentiated cluster levels to enhance construct clarity. Thus, given the potential inconsistencies in how teachers interpret feedback practices in FA, excluding these items at this stage can improve psychometric clarity.

Third, the CFA in the main study not only confirmed the two new sub-dimensions of the *Conceptual* dimension and the revised items in the *Practical* dimension, but also helped refine the *Socio-emotional* dimension into two new sub-dimensions: *Concerning student emotions* and *Respect for students*. The *Concerning student emotions* dimension was a set of the general characteristics of the six items about teachers’ sensitivity of FAL. Stiggins (2002) emphasized the necessity for teachers to possess a heightened sensitivity towards FA both outcomes and procedures. While Leong et al. (2018) highlighted that East Asian teachers often exhibit a strong sense of responsibility for student outcomes, research suggested that under the pressure of high-stakes SA, this responsibility can become utilitarian, leading Chinese teachers to overlook students’ emotional needs (Tan, 2020). Chinese students’ emotions are closely linked to perfectionism, which is deeply rooted in the competitive Confucian culture (Fong and Cai, 2019). Further, Ma et al. (2021) found that Chinese students’ emotions can have a significant impact on their learning motivation. As such, if Chinese teachers maintain a Confucian-style authoritative role that engraves subservience and obedience, they are likely to overlook students’ emotions. Additionally, the *Respect for Students* dimension emphasized individualizing instruction within FA practices, with a strong awareness of students’ dispositions. Generally, building a supportive classroom climate through positive teacher-student interactions benefits students’ learning in the high-pressure atmosphere of Chinese classrooms, which remain largely teacher-dominated (Wang et al., 2018). While both concepts acknowledge the importance of students’ mental well-being, it is crucial to distinguish them as two distinct dimensions, particularly within cultural norms influenced by Confucian values that emphasize unidirectional respect for teachers. In conclusion, the improved CFA fit and the cultural salience of differentiating emotions from respect in Confucian classrooms justify treating them as distinct yet related constructs.

The application of psychometric questionnaires should take cultural influences into account (Yan and Pastore, 2022a); however, this is often difficult for Chinese school administrators or teachers as end users when using instruments developed in Western settings. The *FALQ* directly incorporated such influences through culturally grounded items, making it more user-friendly and valid in the Eastern context such as mainland China. These new dimensions and items are novel for quantitative scales specifically designed for CHC contexts. This adaptation ensured that the questionnaire is both theoretically grounded and practically relevant, capturing the unique challenges and opportunities that teachers in China face in front of FAL development. The *TFAPS* and *TFALS* (Yan and Pastore, 2022a,b) are more oriented toward FA practices, whereas the *FALQ* emphasizes teachers’ literacy, given the current status of limited FA implementation in Chinese classrooms. These three questionnaires can be used in a complementary manner, or the *FALQ* serving as a potential prerequisite. In the largely unexplored area of Chinese teachers’ FAL, this questionnaire can serve as an initial survey tool to capture teachers’ profiles and diagnose their needs for continuing professional learning at the first stage, thereby enabling the purposeful design of teacher training (Yan and Pastore, 2022b).

Moreover, the data were collected from Lingang, Shanghai. Although this area represents one of the most diverse educational settings in China in terms of ongoing reforms, teacher and student recruitment, and flexible policies with relatively minimal jurisdictional constraints, the sample size remains small in relation to China’s vast territory. While the data are not nationally representative, they provide a pragmatic and meaningful testbed for the initial validation of the *FALQ*. Consequently, when applying the *FALQ* in other regions of China or in broader Confucian-influenced contexts, additional cultural and contextual adaptations may be required. Researchers and educators in these contexts are therefore encouraged to adapt the questionnaire to their local settings, as variations in teachers’ FAL and in government reforms and policies related to FA are likely to exist across regions (Yan and Brown, 2021). Such adaptation may involve revising item wording or incorporating context-specific examples to ensure alignment with local educational practices and policy environments. Accordingly, future studies should further examine the applicability and validity of the instrument across diverse Confucian-influenced educational systems to strengthen its generalizability.

Lastly, this study has two limitations. First, the validation of the scale primarily focused on internal structure evidence, including factor analytic results and Rasch-based analyses. Although internal structure validity constitutes a foundational component of scale validation, particularly in the early stages of instrument development (Messick, 1995), other sources of validity evidence were not examined in the present study. Specifically, convergent and discriminant validity could not be assessed due to the lack of well-established and contextually appropriate measures of teachers’ FAL in the Chinese educational context. Additionally, criterion-related validity was not examined, as suitable external criteria or performance indicators aligned with teachers’ FAL were beyond the scope of this study. Validation is an ongoing and cumulative process; therefore, future research should extend the present work by incorporating additional sources of validity evidence, including relations to external variables and measurement invariance across relevant subgroups. Second, emerging artificial intelligence and educational technology tools are reshaping assessment practices, particularly FA in online and blended learning contexts (Zhao et al., 2016). Although these developments were not captured in the present framework, they represent an important direction for future extensions of the *FALQ*.

## Data Availability

The raw data supporting the conclusions of this article will be made available by the authors, without undue reservation.
