# Supplementary material for: Developing and validating teacher formative assessment literacy questionnaire in the high-stakes examination culture: a case of China
Source: Front Psychol. 2026 Feb 11;17:1771941. doi: 10.3389/fpsyg.2026.1771941 (PMC12932453; doi:10.3389/fpsyg.2026.1771941)
Supplement: Supplementary file 1 [file Supplementary_file_1.docx]

**Appendix**

*Formative Assessment Literacy Questionnaire* (*FALQ*)

|  | 1 | 2 | 3 | 4 | 5 |
| --- | --- | --- | --- | --- | --- |
| Conceptual: Teachers’ self-efficacy  Q1: I know how to clarify formative assessment criteria and goals with students.  Q2: I understand how to organize a series of formative assessment activities.  Q3: I care about the effectiveness of my formative assessment practices.  Q4: I know diverse formative assessment methods that allow students to demonstrate their learning. | | | | | |
| Conceptual: Knowledge for collaboration with students  Q5: I understand what feedback is useful for students.  Q6: I consider students’ feelings when I provide feedback.  Q7: I think students and peers should be motivated in the formative assessment process.  Q8: I believe formative assessment tasks are useful for modifying teaching and learning.  Q9: I know how to motivate students’ learning by proper use of formative assessment methods.  Q10: I understand the power relationship between students and me is equal in formative assessment. | | | | | |
| Practical (revised) | | | | | |
| Q11: I use various assessment activities in the classroom to elicit information of students’ learning.  Q12: I design my formative assessment activities into my teaching outline.  Q13: I provide different feedback for different scenarios after formative assessment. (Removed)  Q14: I encourage students to discuss, respond, and argue with my feedback.  Q15: I share assessment rationale with students and may modify it building on students’ ideas.  Q16: I show and explain the gap between formative assessment outcomes and criteria.  Q17: I provide further suggestions for students to improve their performance in the end. (Removed)  Q18: I guide students to take part in peer feedback. (Removed)  Q19: I instruct students to identify strengths and weaknesses via formative assessment. (Removed)  Q20: I encourage students to compare their performances at different stages of their own work. (Removed) | | | | | |
| Socio-emotional: Concerning student emotions | | | | | |
| Q21: I encourage students to bravely express self-feelings, opinions or attitudes in formative assessment process.  Q22: I can perceive student emotions through the process of formative assessment. Q23: I recognize student backgrounds may impact the effectiveness of formative assessment.  Q24: I am careful not to negatively influence student learning in my feedback.  Q26: I use different methods to trigger students’ positive attitude towards formative assessment.  Q27: I organize students to make appropriate peers voluntarily or deliberately. | | | | | |
| Socio-emotional: Respect for students  Q25: I respect students while communicating all the formative assessment aspects.  Q28: I am sensitive to the privacy of students in the process of formative assessment.  Q29: Both students and I are responsible for the implementation of formative assessment.  Q30: I communicate the formative assessment results with parents and students. | | | | | |
